# Beyond hypertrophy: four-chamber myocardial involvement in ATTR cardiac amyloidosis diagnosed by multimodality imaging

**DOI:** 10.21542/gcsp.2026.31

**Published:** 2026-08-31

**Authors:** Sridevi Chigullapalli, Ajitkumar Jadhav, Susheel Kumar Malani, Sravanth Gandavarapu

**Affiliations:** Dr D Y Patil Medical College Hospital and Research Centre, Pune, Maharashtra, India

## Abstract

**Background:** Cardiac amyloidosis results from the deposition of amyloid fibrils in the myocardial extracellular matrix, leading to restrictive cardiomyopathy and heart failure. Diagnosis has traditionally been challenging owing to non-specific clinical features and reliance on endomyocardial biopsy. Advances in cardiovascular imaging have, however, improved early detection.

**Case summary:** We present a 74-year-old man with progressive dyspnea on exertion and bilateral pedal edema of three months’ duration. Cardiac amyloidosis was suspected on the basis of discordance between electrocardiographic and echocardiographic findings, and was confirmed using global longitudinal strain (GLS), cardiac magnetic resonance imaging (CMR), and bone scintigraphy.

**Conclusion:** This case underscores the importance of recognizing cardiac amyloidosis as an underdiagnosed cause of heart failure. Modern imaging modalities facilitate non-invasive, early diagnosis, often obviating the need for biopsy. Early identification is crucial, as emerging therapies offer the potential to modify disease progression and improve outcomes in a condition once considered incurable.

## Introduction

Cardiac amyloidosis, characterized by the extracellular deposition of misfolded amyloid fibrils within the myocardium, is an important cause of restrictive cardiomyopathy and heart failure. The two principal types are immunoglobulin light-chain (AL) amyloidosis and transthyretin (ATTR) amyloidosis, the latter further classified into wild-type (ATTRwt) and hereditary/variant (ATTRv) forms^3,4,6^.

Previous studies have focused on improving diagnostic accuracy and exploring novel therapies aimed at limiting further amyloid deposition and modifying disease progression. Despite these advances, cardiac amyloidosis remains underdiagnosed and is frequently mistaken for other causes of heart failure. The classic presentation comprises heart failure—typically with preserved ejection fraction—together with conduction abnormalities and characteristic multimodality imaging findings.

We describe a case in which discordance between electrocardiographic and echocardiographic findings prompted a multimodality evaluation, revealing infiltrative disease extending well beyond the wall-thickening phenotype on which the diagnosis is often anchored.

## Case presentation

### Chief complaints and history

A 74-year-old man presented to the cardiology department with exertional dyspnea (NYHA class II) that had progressed gradually over three months. He also reported bilateral pitting pedal edema extending to the knees, present for 15 days. He was admitted for further evaluation and management.

### Past medical history

The patient had a known history of type 2 diabetes mellitus and hypertension, both diagnosed six months earlier and under regular treatment. He had benign prostatic hyperplasia, for which transurethral resection of the prostate (TURP) had been performed six months before presentation.

### Physical examination

On admission, the patient was hemodynamically stable. There was no pallor, icterus, clubbing, or cyanosis. Systemic examination revealed bilateral pitting pedal edema extending to the knees. Cardiovascular examination demonstrated normal first and second heart sounds (S1 and S2) with no murmurs or gallops. Respiratory examination revealed equal air entry bilaterally with bibasal crepitations. The abdomen was unremarkable. The patient was conscious and oriented, with a Glasgow Coma Scale of 15/15.

### Electrocardiography

**Figure 1.**
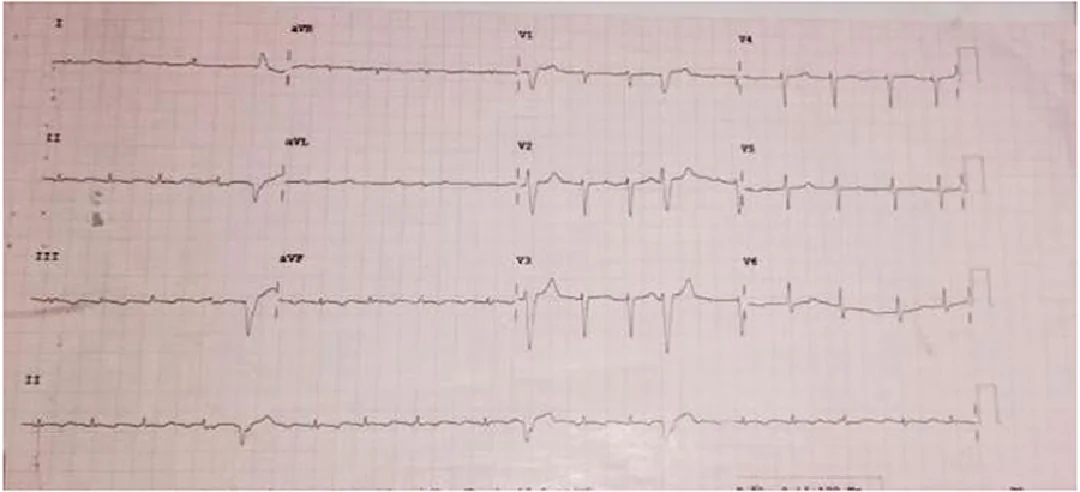
Electrocardiogram showing atrial fibrillation with low voltage limb leads and poor R wave progression.

The ECG demonstrated atrial fibrillation with low-voltage limb leads and poor R-wave progression—findings highly suggestive of infiltrative cardiac disease (Figure 1).

### Transthoracic echocardiography

**Figure 2.**
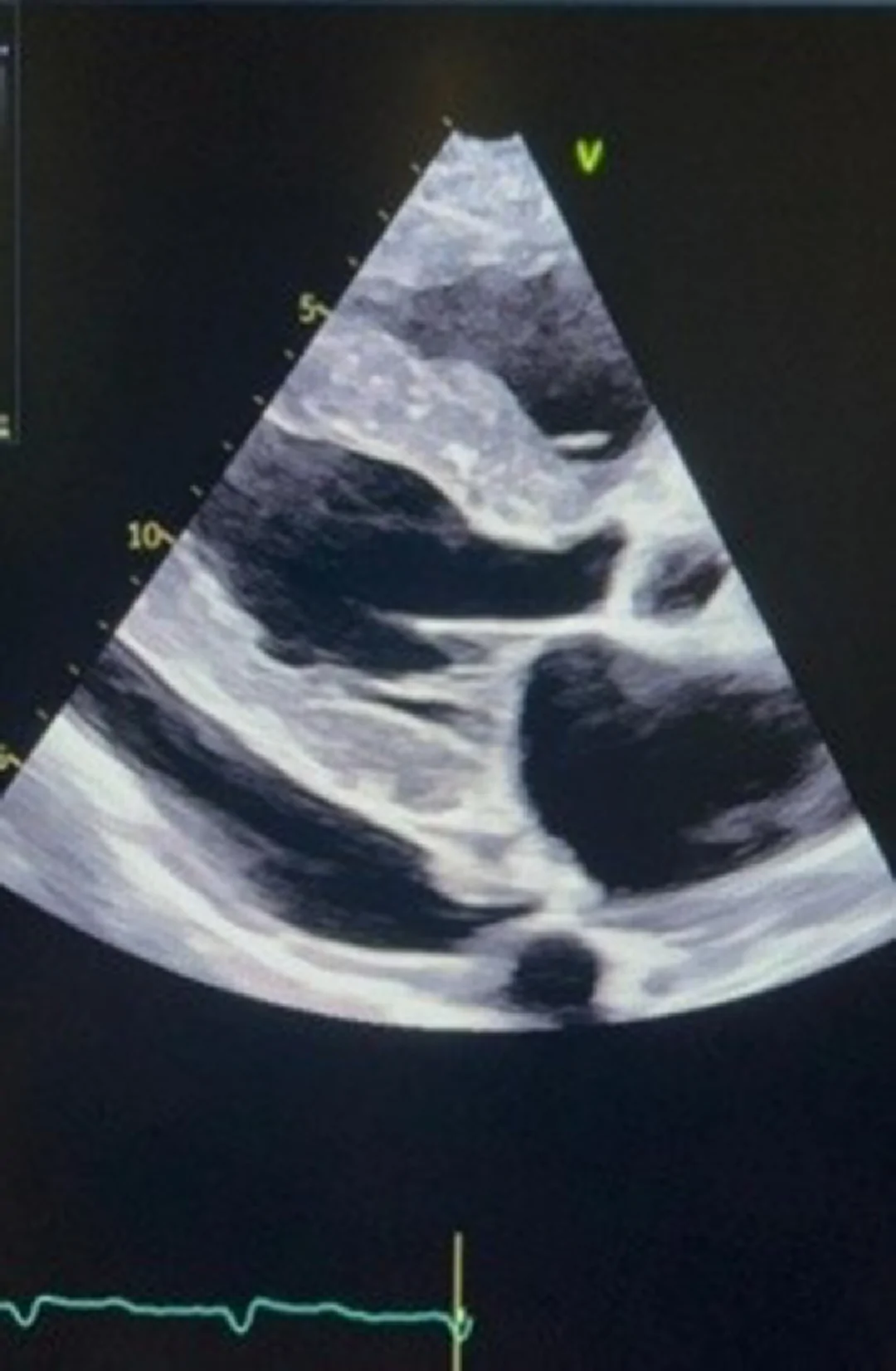
Parasternal long axis view showing dilated left atrium along with LVH and pericardial effusion.

**Figure 3.**
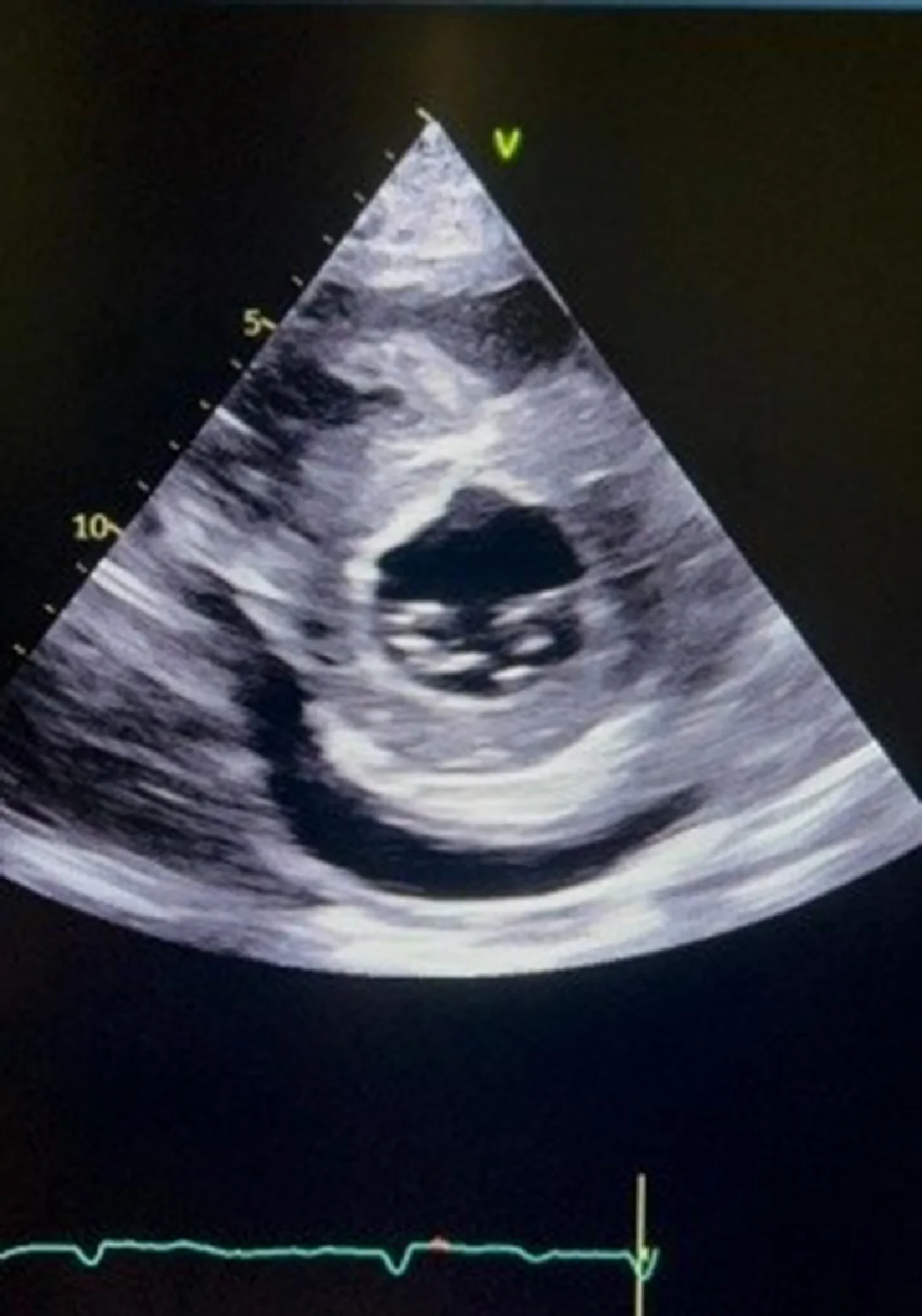
Short axis view showing effusion and ventricular hypertrophy.

**Figure 4.**
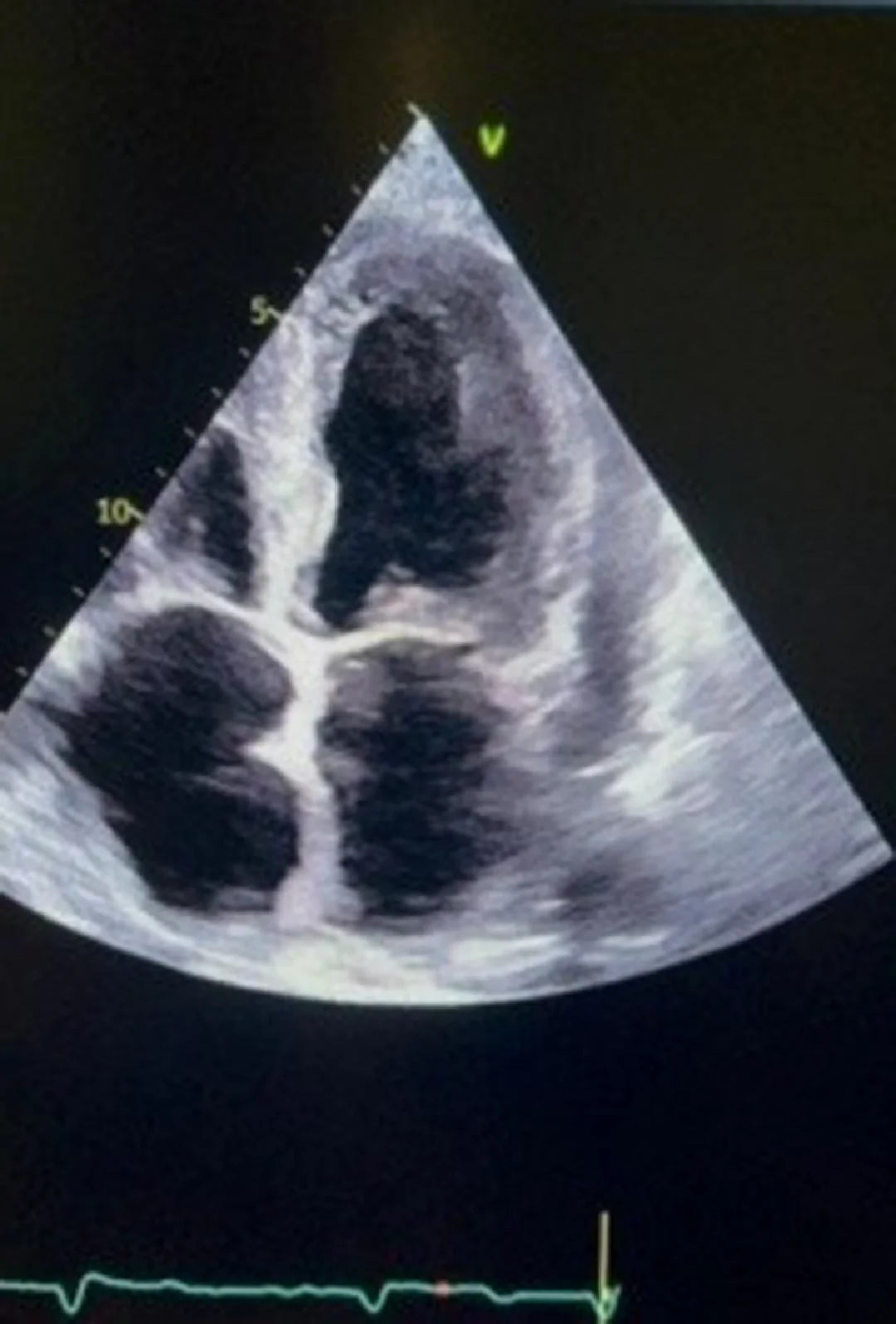
Apical 4-chamber view showing bi-atrial enlargement with ventricular hypertrophy and mild dilatation.

**Figure 5.**
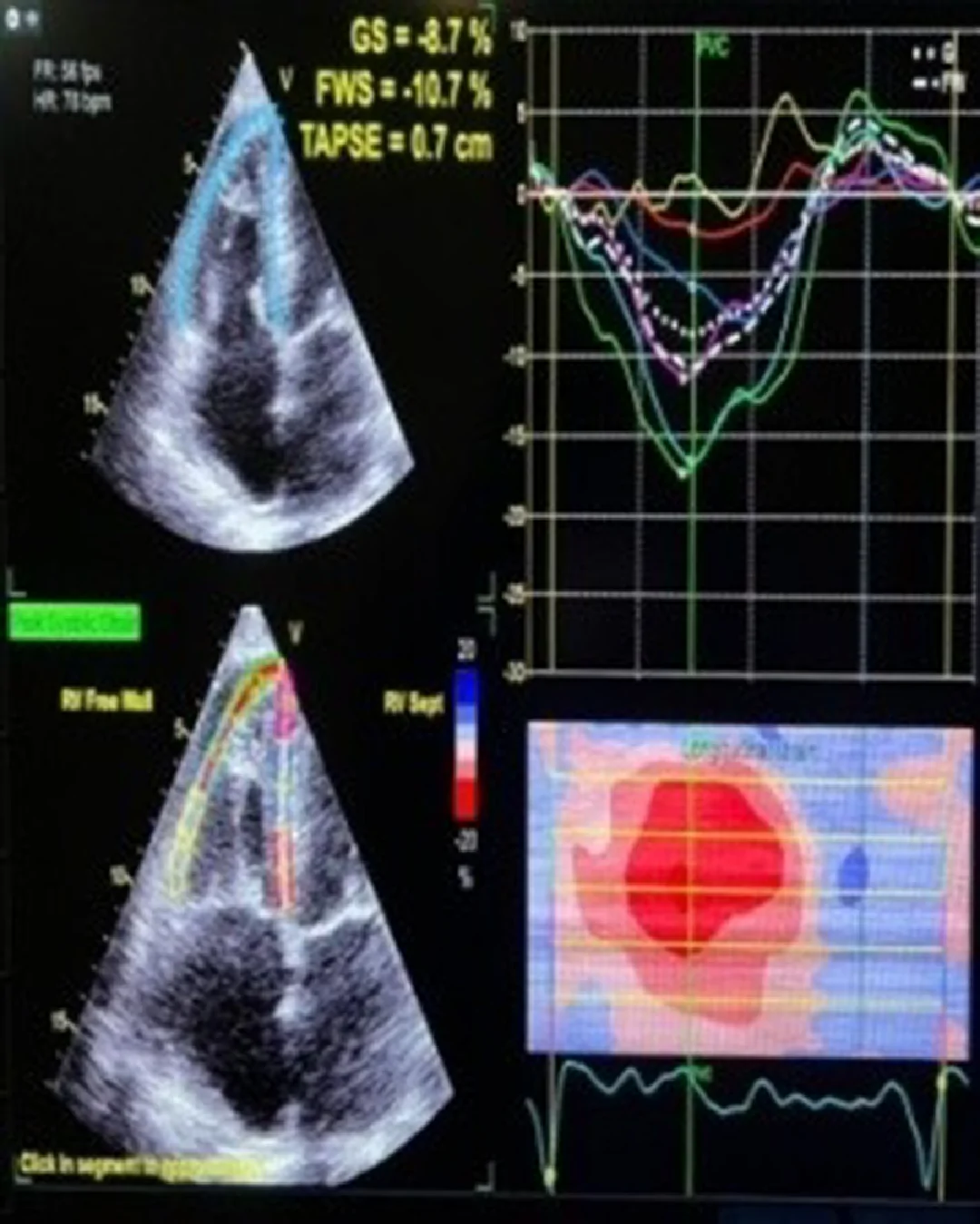
GLS of RV shows RV strain of −8.7% with TAPSE –0.7 cm.

**Figure 6.**
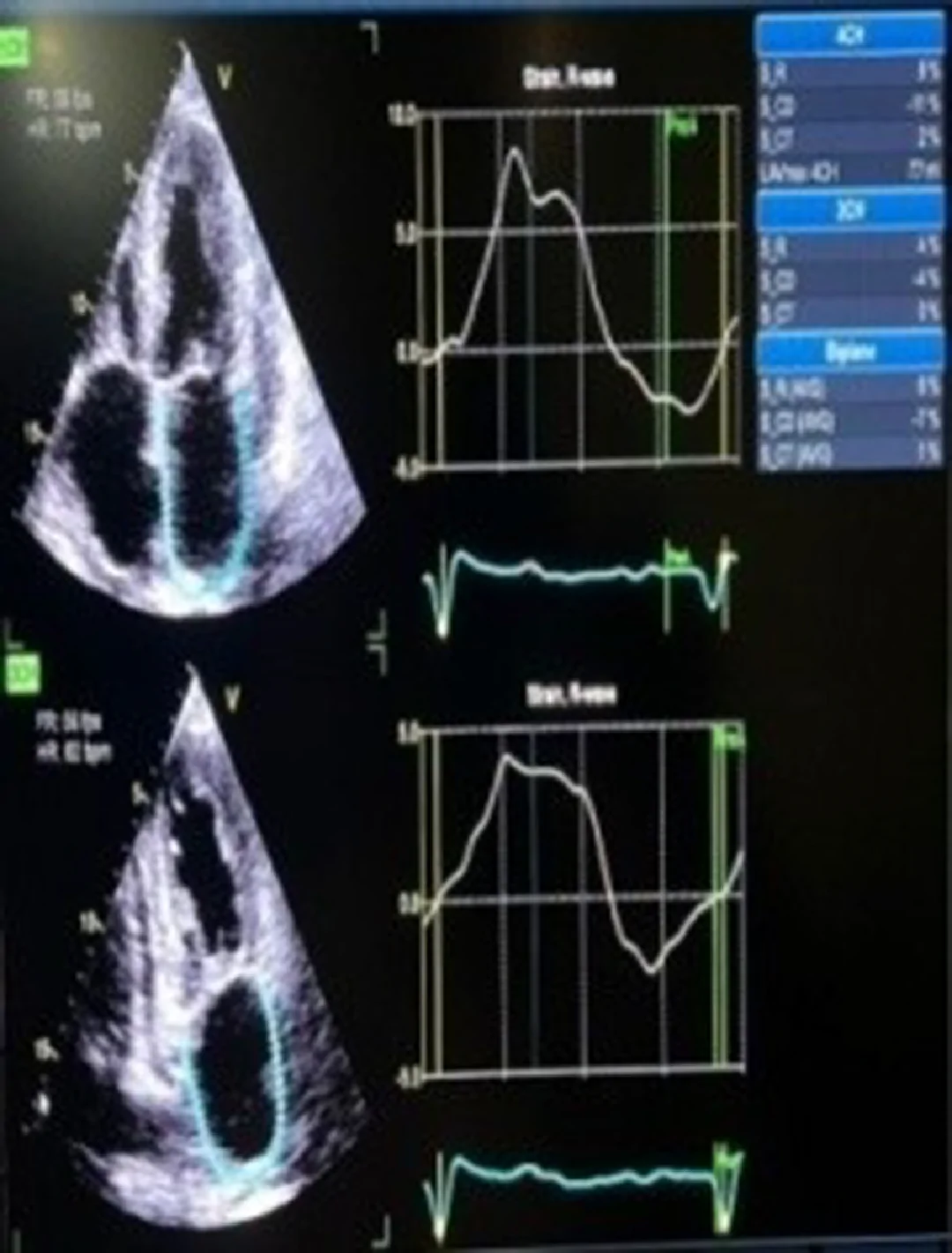
LA Strain imaging. 4CH - Reservoir Strain- 8%, Conduit Strain-11% 2CH - Reservoir Strain- 4%, Conduit strain- 4%.

**Figure 7.**
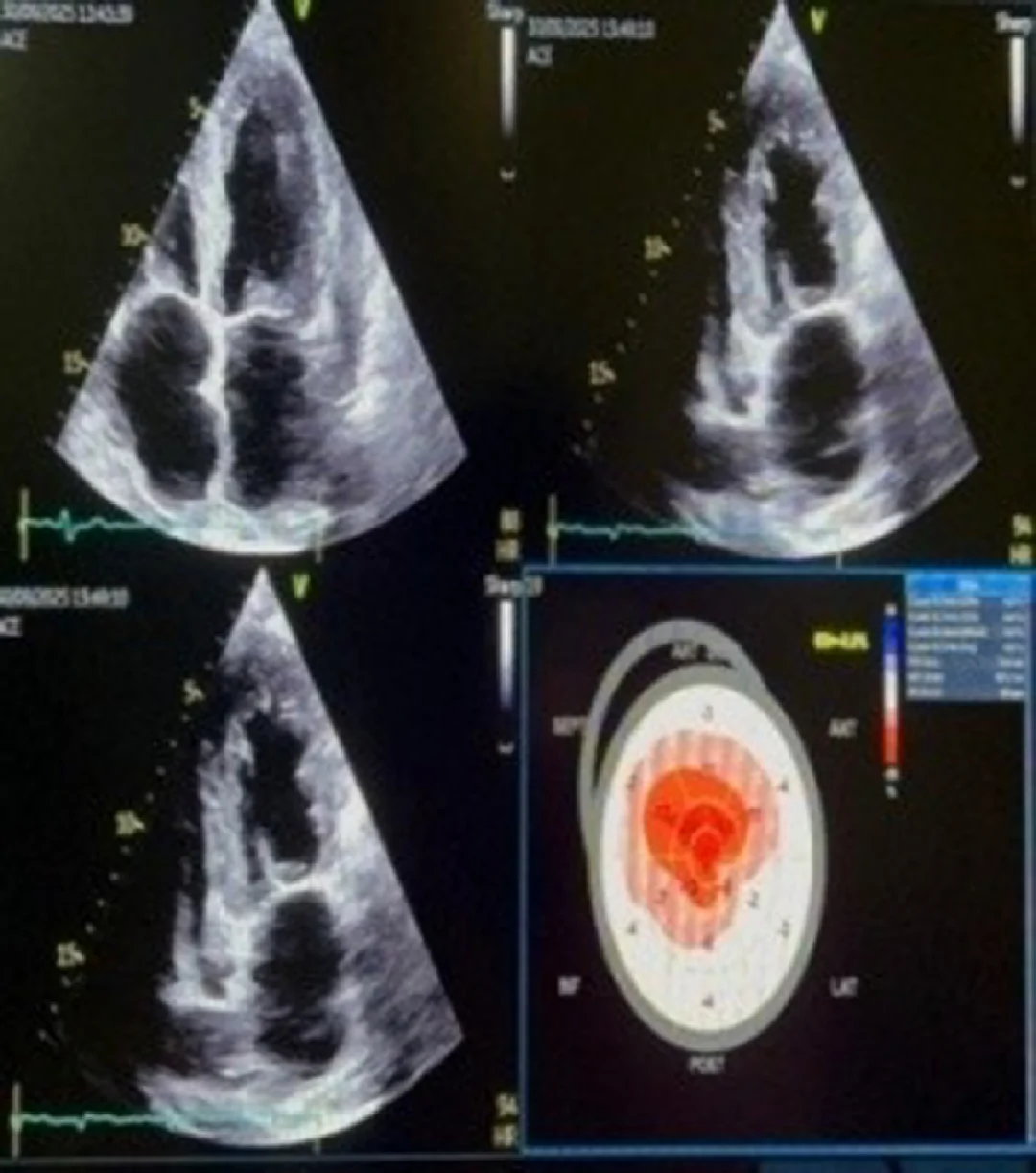
GLS of LV shows LV strain of −8.8% with cherry on top appearance (apical sparing).

Transthoracic echocardiography revealed severe left ventricular (LV) systolic dysfunction, with an ejection fraction of 25–30% by visual estimation, concentric LV wall thickening, biatrial dilatation, and mildly dilated left and right ventricles. There was grade 2 diastolic dysfunction. Valvular assessment showed moderate mitral regurgitation, trivial aortic regurgitation, and mild tricuspid regurgitation, with no stenotic lesions. Strain imaging demonstrated relative apical sparing (the “cherry-on-top” pattern) on the bull’s-eye map, with severely reduced global longitudinal strain (LV GLS −8.8%; RV free-wall strain −8.7%). A moderate pericardial effusion was noted, and the inferior vena cava measured 15 mm with normal respiratory collapse. Left atrial strain analysis showed reduced reservoir, conduit, and contractile function (Figures 2–7).

### Cardiac magnetic resonance imaging

**Figure 8.**
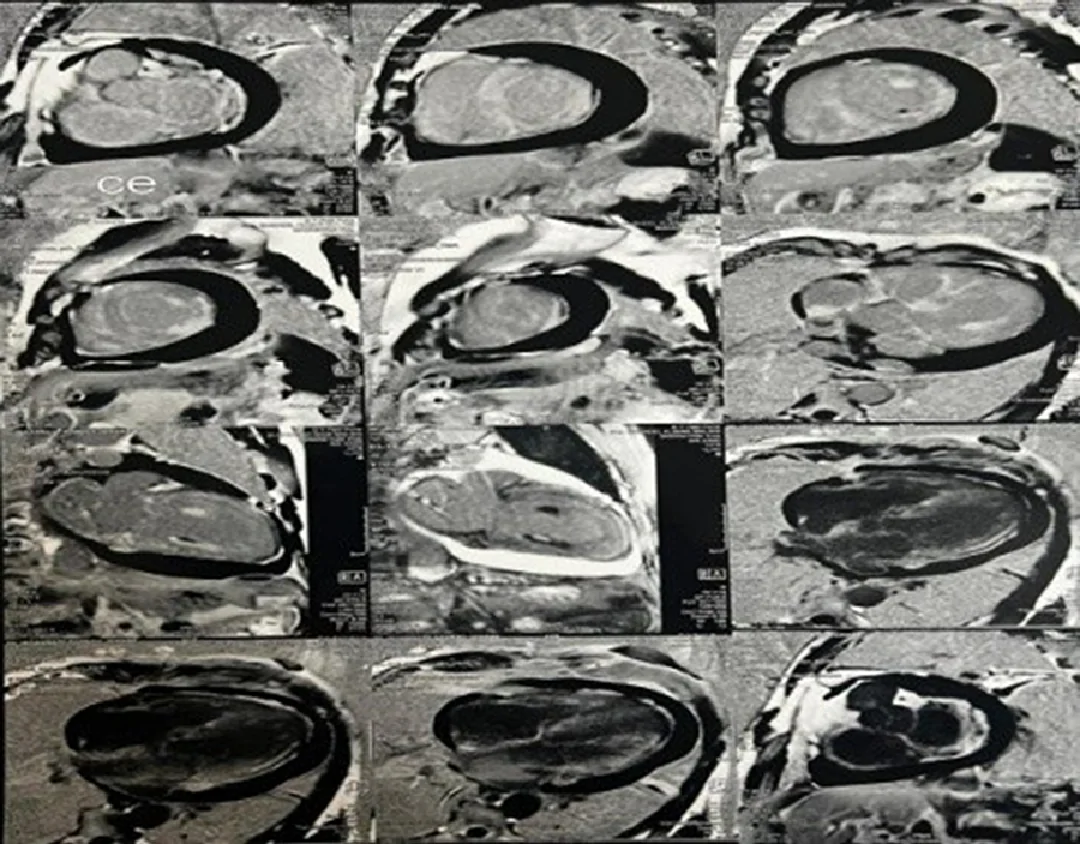
Cardiac magnetic resonance imaging (CMR) of transthyretin cardiac amyloidosis. Representative late gadolinium enhancement (LGE) images obtained in long-axis and short-axis planes demonstrate diffuse concentric left ventricular wall thickening, biatrial enlargement, and extensive circumferential subendocardial LGE, consistent with diffuse myocardial amyloid infiltration.

**Figure 9.**
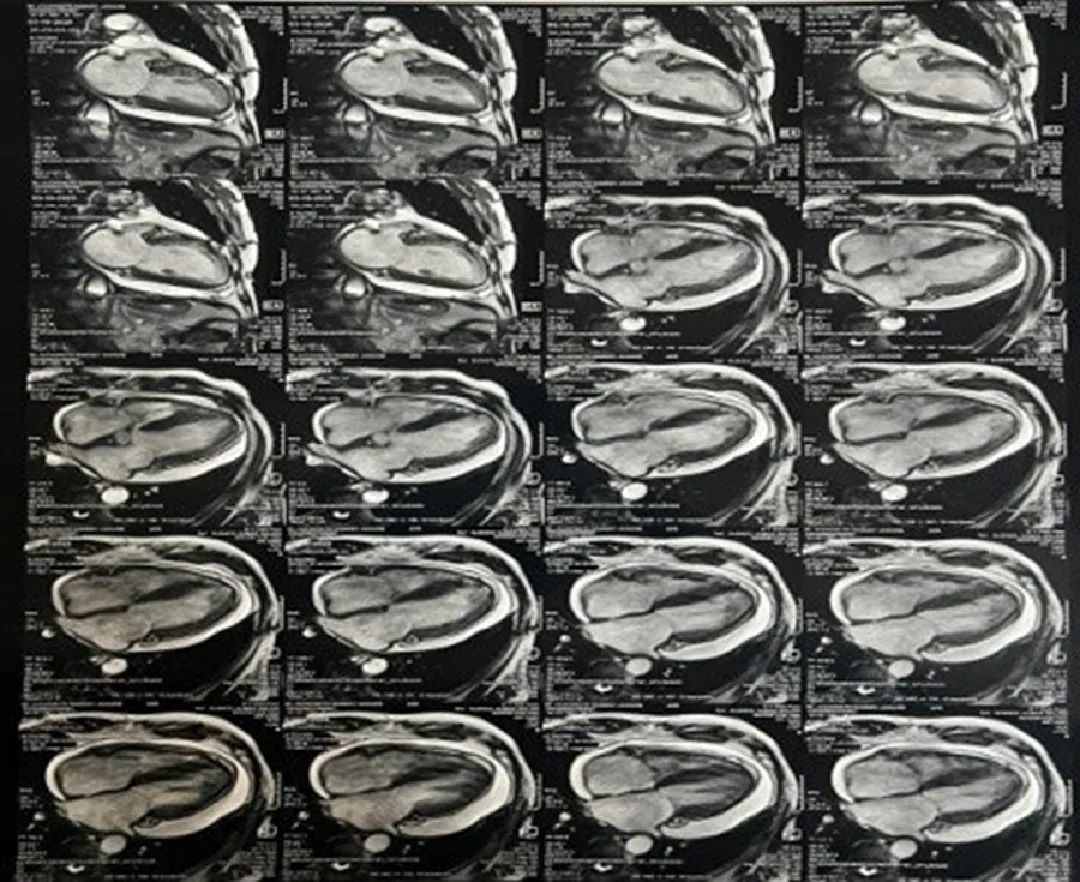
Cine-balanced steady-state free precession (bSSFP) CMR images in sequential four-chamber views demonstrate concentric left ventricular hypertrophy, biatrial enlargement, and diffuse myocardial thickening.

**Figure 10.**
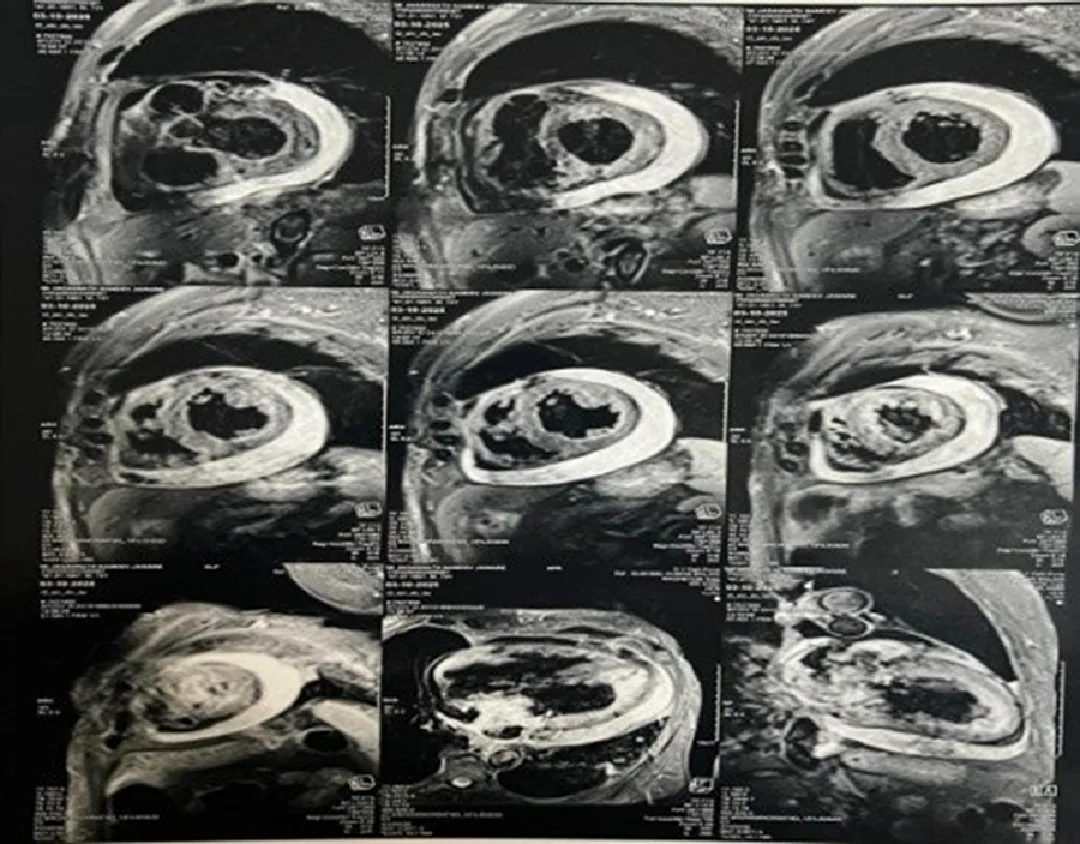
Cine balanced steady-state free precession (bSSFP) CMR images in sequential short-axis views from the basal to apical left ventricle. Demonstrating diffuse concentric LV wall thickening and preserved myocardial geometry, consistent with an infiltrative cardiomyopathy.

CMR revealed mildly dilated left and right ventricles, with ejection fractions of 32% and 25% respectively, and biatrial dilatation. TI-scout (Look-Locker) images showed abnormal myocardial nulling, with the myocardium nulling before the LV blood pool—a characteristic feature of cardiac amyloidosis. Perfusion imaging demonstrated hypoperfusion involving the papillary muscles. Late gadolinium enhancement was near-circumferential and near-transmural, extending from base to apex of the left ventricle and involving the right ventricular free wall, both atria, and the papillary muscles; a moderate pericardial effusion was again noted. These features were consistent with an infiltrative cardiomyopathy affecting all four cardiac chambers, in keeping with amyloidosis (Figures 8–10).

**Figure 11.**
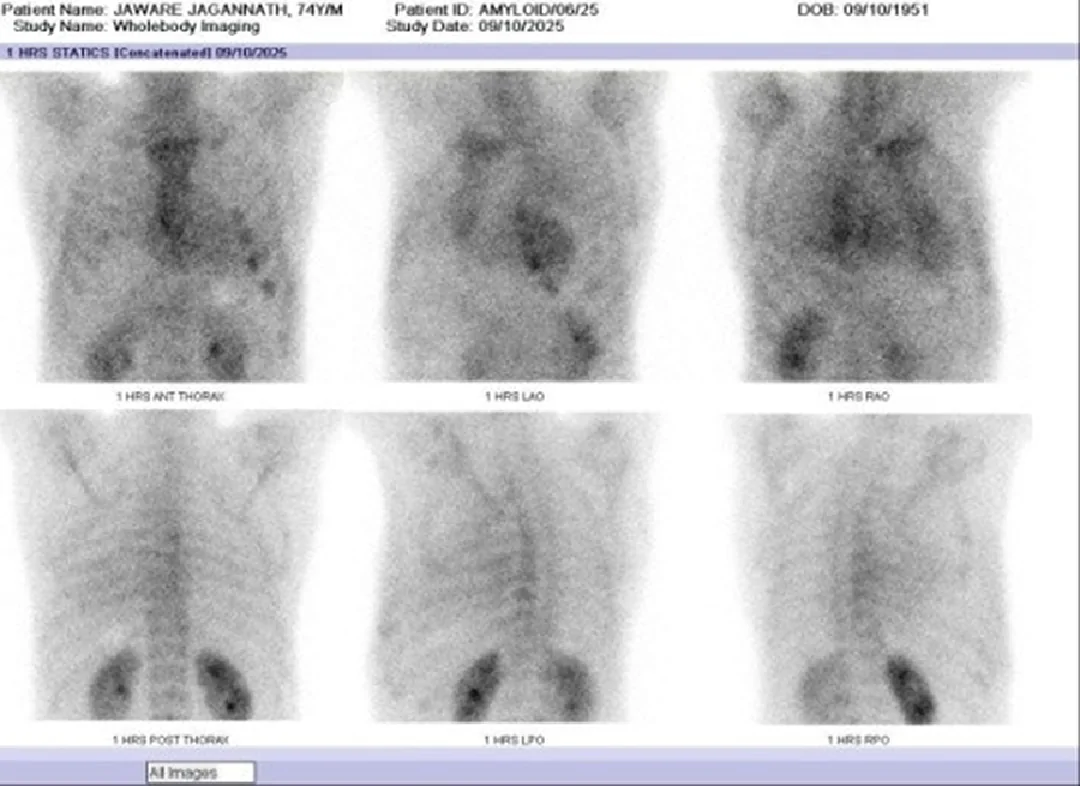
Planar 99mTc-pyrophosphate (99mTc-PYP) scintigraphy performed 1 h after tracer administration. Anterior, left anterior oblique (LAO), right anterior oblique (RAO), posterior, left posterior oblique (LPO), and right posterior oblique (RPO) thoracic projections demonstrate diffuse, intense myocardial tracer uptake exceeding adjacent rib uptake (Perugini grade 3), highly suggestive of transthyretin cardiac amyloidosis.

**Figure 12.**
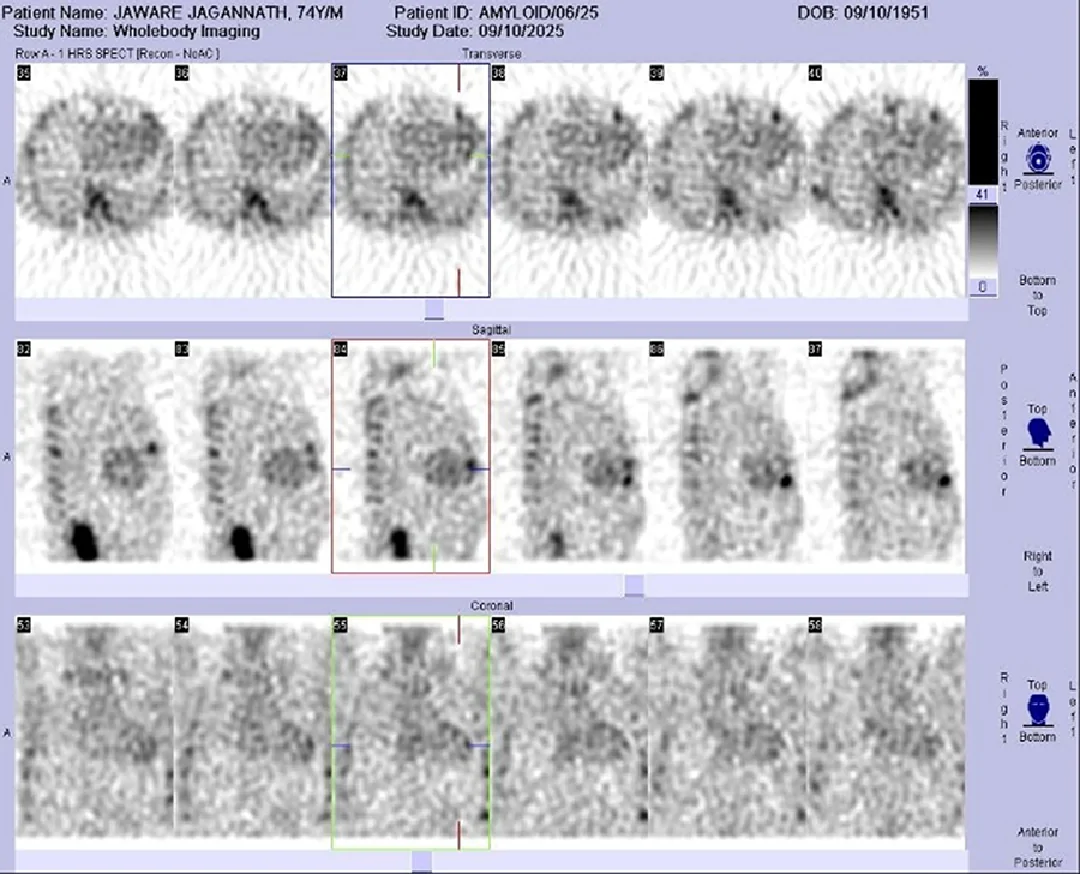
Single-photon emission computed tomography (SPECT) images acquired 1 h after 99mTc-pyrophosphate administration. Transverse, sagittal, and coronal reconstructions demonstrate diffuse radiotracer localization within the left ventricular myocardium, confirming true myocardial uptake and excluding blood-pool activity.

**Figure 13.**
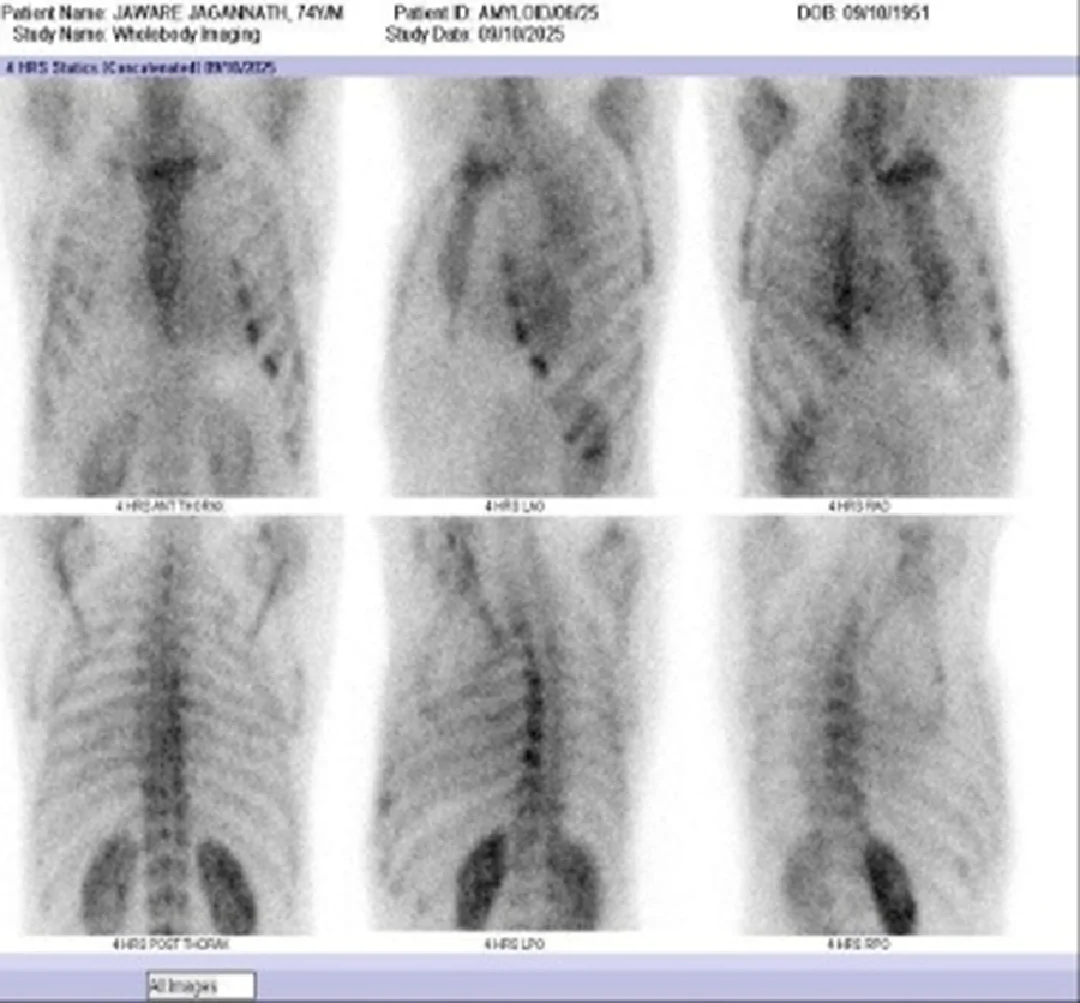
Delayed (4-hour) planar 99mTc-pyrophosphate scintigraphy. Anterior, left anterior oblique (LAO), right anterior oblique (RAO), posterior, left posterior oblique (LPO), and right posterior oblique (RPO) thoracic images demonstrate persistent diffuse myocardial radiotracer uptake with reduced blood-pool activity.

### ^99_m_^Tc-pyrophosphate scintigraphy

^99_m_^Tc-pyrophosphate (PYP) scintigraphy demonstrated diffuse myocardial tracer uptake exceeding rib uptake, corresponding to Perugini grade 3, with a heart-to-contralateral-lung ratio of 1.52. In the absence of a detectable monoclonal protein, these findings were diagnostic of ATTR cardiac amyloidosis without the need for endomyocardial biopsy^1,5^ (Figures 11–13).

### Diagnosis and management

On the basis of the ECG, echocardiographic, CMR, and ^99m^Tc-pyrophosphate findings, together with the patient’s age, absence of a family history, and predominantly cardiac involvement, a diagnosis of wild-type ATTR cardiac amyloidosis (ATTRwt) was made.

### Treatment

Management was directed at two objectives:

1. **Disease-specific therapy.** Tafamidis 61 mg once daily—a transthyretin tetramer stabilizer shown to reduce cardiovascular mortality and hospitalization in ATTR cardiomyopathy^2^—was recommended, but the patient declined owing to the cost of the medication.
2. **Heart failure management.** Guideline-directed medical therapy for heart failure with reduced ejection fraction was continued, alongside symptomatic treatment.

## Discussion

This case illustrates several features of the diagnosis and management of cardiac amyloidosis in the modern era. The patient presented with relatively rapid symptom progression over three months and, at presentation, involvement of all four cardiac chambers. The diagnosis was first suspected from the classic discordance between low-voltage ECG and increased wall thickness on echocardiography, together with an apical-sparing pattern of longitudinal strain.

The extent of myocardial involvement in this case represents a severe form of the disease. The CMR findings—near-circumferential, near-transmural late gadolinium enhancement from base to apex, with biventricular and biatrial involvement—reflect the widespread distribution of amyloid deposition^7^ and give substance to the observation that infiltration extends well beyond the wall-thickening phenotype on which the diagnosis is often anchored.

^99_m_^Tc-pyrophosphate scintigraphy was central to the diagnostic pathway. Grade 3 myocardial uptake with a heart-to-contralateral-lung ratio of 1.52, in the absence of a monoclonal protein, provided non-invasive confirmation of ATTR cardiac amyloidosis and avoided endomyocardial biopsy and its attendant risks. This reflects a broader shift in practice, in which a validated combination of non-invasive tests can establish the diagnosis in appropriately selected patients.

The emergence of disease-modifying therapy, notably the transthyretin stabilizer tafamidis, has changed the outlook in ATTR cardiac amyloidosis from a uniformly progressive condition to one in which meaningful intervention is possible—although, as this case shows, access can be limited by cost.

## Limitations

Wild-type ATTR designation was based on the patient’s age, the absence of a known family history, and predominant cardiac involvement rather than on TTR genotyping, which was not performed; hereditary ATTR can also present at an advanced age with cardiac-predominant disease and without an apparent family history, and genetic testing would be required for definitive classification.

Although AL amyloidosis workup was negative, it cannot be excluded entirely.

The patient declined disease-modifying therapy owing to financial constraints, no data on treatment response or longer-term outcome are available.

## What have we learned?

Cardiac amyloidosis remains an under-recognized cause of heart failure. A discordance between ECG voltage and echocardiographic wall thickness should prompt dedicated evaluation, and a structured multimodality pathway—strain echocardiography, CMR, and bone scintigraphy with monoclonal-protein exclusion—can establish the diagnosis non-invasively. Early identification is increasingly consequential, as disease-modifying therapy can now alter the course of what was once considered untreatable.
